# Oral human papillomavirus infections in Zambian Rural and Urban residents-a community cross-sectional study

**DOI:** 10.1186/s12903-024-05312-4

**Published:** 2024-12-22

**Authors:** Chrispinus Hakimu Mumena, Schifra Uwamungu, Göran Kjeller, Bengt Hasséus, Maria Andersson, Daniel Giglio

**Affiliations:** 1 Department of Oncology, Institute of Clinical Sciences, Sahlgrenska Academy at the University of Gothenburg, Gothenburg, Sweden; 2 Department of Dental Clinical Sciences, School of Medicine, Copperbelt University, Ndola, Zambia; 3 Department of Pharmacology, Institute of Neuroscience and Physiology, Sahlgrenska Academy at the University of Gothenburg, Gothenburg, Sweden; 4 Department of Biomedical Laboratory Sciences, School of Health Sciences, College of Medicine and Health Sciences, University of Rwanda, Kigali, Rwanda; 5 Department of Oral and Maxillofacial Surgery, Institute of Odontology, Sahlgrenska Academy at the University of Gothenburg, Gothenburg, Sweden; 6 Department of Oral Medicine and Pathology, Institute of Odontology, Sahlgrenska Academy at the University of Gothenburg, Gothenburg, Sweden; 7 Department of Infectious Diseases, Institute of Biomedicine, Sahlgrenska Academy at the University of Gothenburg, Gothenburg, Sweden; 8 Department of Oncology, Sahlgrenska University Hospital, Gothenburg, Sweden

**Keywords:** Oral, HPV, Africa, Orogenital, Sex

## Abstract

**Background:**

How common it is with the presence of human papillomavirus (HPV) in the healthy and diseased oral cavity is largely unknown for Africans. In this cross-sectional study we assessed the prevalence of oral HPV and the risk factors associated with HPV contraction including sexual practice in the urban and rural Zambian population.

**Methods:**

Urban (*N* = 188) and rural (*N* = 211) Zambian adults aged 21 years and older living in Ndola and Mansa, respectively, were interviewed about demographical data, oral and coital sexual history and tobacco and alcohol use. Participants were orally examined and underwent a buccal swab test for 12 high-risk HPVs (HPV16, 18, 31, 33, 35, 39, 45, 51, 52, 56, 58, and 59) and two low-risk HPVs (HPV6 and 11) with real-time PCR.

**Results:**

Alcohol consumption was higher in urban participants than rural participants, i.e., 34.1% and 16.6%, respectively, consumed alcohol once a week or more (*p* = 0.001). Ever-smokers constituted 38.8% of urban and 32.2% of rural participants (*p* = 0.363). Engaging in orogenital sex was uncommon, however, more common in urban than rural participants (13.3% and 4.3%, respectively, *p* = 0.003). Only three participants were positive for HPV (HPV16, 35, and 45, respectively).

**Conclusions:**

Urban participants displayed higher sexual risk behaviour than rural participants. However, the prevalence of oral HPV infection in Zambia was low, which contrasts to the high incidence of cervical cancer reported for the country.

**Supplementary Information:**

The online version contains supplementary material available at 10.1186/s12903-024-05312-4.

## Background

Human papillomavirus (HPV) infection is the most common sexually transmitted infection affecting mucous membranes of the genital, anal and oral tracts. The HPV family consists of over 200 different HPV types where some of them may potentially contribute to cancer development, i.e., high-risk (HR-) HPV types are considered the HPV16, 18, 31, 33, 35, 39, 45, 51, 52, 56, 58, and 59 and most likely also HPV68 [1]. HPV is close to 100% associated with cervical cancer and it is the fourth most common cancer form in the world [2]. HPV is also associated with many other cancer forms including cancers of the oral cavity. HPV-associated cancer of the oral cavity occurs predominantly in the tonsils and base of tongue where HPV16 is the dominant HPV type [3]. We are currently seeing an increase in HPV-associated oropharyngeal squamous cell carcinoma (OPSCC), particularly in the western world [4]. HPV-associated OPSCC accounts for example for 85% of all OPSCC in Sweden [5]. The association between HPV infection and oral potentially malignant disorders (OPMDs) and oral squamous cell carcinoma (OSCC) is less strong. In a multicenter study conducted in Sweden, Romania and Brazil, we showed that HR-HPV was present in 2% of oral leukoplakia cases from Brazil and in no cases from Romania or Sweden [6]. The prevalence of HR-HPV in OSCC varies a lot between studies, regions in the world and in the methodology used to detect HPV with the random proportion of 20.1% shown in a meta-analysis [7]. Transmission of HPV between different mucous membranes during sex is common. Increased orogenital sex practice is considered as the main cause of the increased incidence in HPV-associated OPSCC in western countries during the last three decades [8]. Similar to HPV infections of the uterine cervix, studies demonstrate that HPV infections may be persistent for years also in the oral cavity, particularly in people living with HIV [9]. This may constitute a risk factor for the development of OPSCC.

Cervical cancer stands for the predominant cause of female cancer deaths in sub-Saharan Africa including Zambia [2]. The prevalence of HPV-associated cancer forms besides cervical cancer has been studied to a little degree in sub-Saharan Africa above South Africa. The natural occurrence of HPV in the oral cavity of healthy individuals in Africa is largely unknown. We showed that 10–12% of Rwandan men and women living with HIV under antiretroviral therapy were infected by HR-HPV [10]. The natural occurrence of HPV in the general population is unknown for most countries in Africa. In view of cervical cancer being so prevalent in this region of the world the aim of this study was to explore the prevalence of oral HPV infections among the rural and urban populations in Zambia and risk factors associated with HPV contraction.

## Methods

### Ethical approval

was obtained from the Tropical Disease Research Centre (TDRC) Institution Review Board (IRB) followed by recommendation from the National Health Research Authority (NHRA) and from the Swedish Ethical Review Authority (2021–02464). The present cross-sectional study was conducted in the rural Mansa and Ndola urban areas in Zambia. Mansa is a district in the Luapula Province, and it has a population of 205,000 inhabitants, whereas 127,000 live in the rural area. The district has 16 wards where the rural wards Chinsuka and Lubende were selected for the study by simple random sampling. Ndola is a district in the Copperbelt Province and has a population of 375,000 inhabitants. Ndola is divided into 28 wards where simple random sampling was used to choose the urban Dag Hammarskjöld ward. The rural and urban areas were selected by the cluster sampling method (rural vs. urban) and the two districts chosen were selected by the stratified sampling method considering alcohol and tobacco use, prevalence of head and neck cancer and low transit activity. Data collection involved mobilization of the community through church leaders and priests, while in the residential and work areas the announcement for the study was made through public announcement with the use of mega speakers in an open car and on foot house to house in places cars could not access. In each zone, the community members were invited to gather at well-known outreach sites/centers frequently used by health centers in the district. These centres are well known in the community for outreach and research mobilization activities related to health.

The study recruited a total of 399 study participants, which had come to the outreach sites/centers. This sample size was obtained by estimating the marginal error and precision to 5%, population proportion of HPV infection to 50% and the non-response rate to 2%. The study population consisted of adults aged 21 years and older. After obtaining informed consent, 188 participants from Ndola and 211 participants from Mansa were recruited. Participants were interviewed and a questionnaire was filled out (supplementary file). Confidentiality was assured during the interview process. The registered variables were age, sex, residential area, marital status, education status, employment status, oral and coital sexual history, HIV status, oral hygiene status, medical diseases suffered in the past, tobacco use and alcohol intake habits. The interview was followed by an oral examination and the buccal mucosa was swabbed for HPV using the Aptima Multitest Swab (Hologic Inc., Marlborough, MA, USA) followed by placing the swabs in the transport media of the tubes.As per standard instruction, city and country are required for affiliations; however, the city and country information was missing in all affiliations. Please check if the provided cities and countries are correct and amend if necessary.We have rectified some of the affiliations. Please note that I mistakenly may have changed the online link and link to the supplemental materials. Please check so it is correct!

### Real-time PCR for HPV detection

Nucleic acid from 500 µL specimen was extracted in a MagNA Pure LC instrument (Roche Diagnostics, Mannheim, Germany), by using external lysis program for the the DNA Isolation I kit. The extracted nucleic acids were eluted in a volume of 100 µl, and 5 µl were used for each PCR reaction. The real-time PCR, targeting 12 HR-HPVs (HPV16, 18, 31, 33, 35, 39, 45, 51, 52, 56, 58, and 59), two low-risk HPVs (HPV6 and 11), and beta-globin, was performed in 8 parallel 20 µl reactions, containing oligonucleotides and Universal Mastermix (Applied Biosystems, USA). Amplification (15 s at 95 °C, 60 s at 58 °C) was run for 45 cycles after an initial 10-minute denaturation at 95 °C in a QuantStudio 6 (Applied Biosystems, Waltham, MA, USA). For more detailed information about the method and primer and probe sequences, we refer to previously published descriptions [5, 11].

### Statistics

The χ^2^-test was used to compare categorical variables between urban and rural participants. The mean value ± standard error of the mean (SEM) is given in the text. Statistical significance was set at a p-value less than 0.05. IBM SPSS Statistics version 26 (IBM Corp., Armonk, NY, USA) and GraphPad Prism program 9.1.0 (GraphPad Software, Inc., San Diego, USA) were used to analyze the data.

## Results

### Sociodemographic data

Table 1 displays the frequency distribution of study participants according to their socio-demographic profile. Women constituted 56% of the studied participants and the majority of the total study population of men and women were young adults (59%). The age range was 21 to 92 years, and the mean age was 39 ± 0.7 years (*n* = 399). In the rural area, women constituted 61.6% of the study participants, while in the urban area women and men were almost 1 to 1. In the rural area, 45.5% had not completed primary school compared to 28.2% in the urban area (*p* < 0.0001). About one third of the participants, both in the urban and rural areas, were affected by chronic diseases, such as cardiovascular diseases, rheumatic diseases, cancer, and other serious not specified diseases. For the urban study population, 11.2% lived with HIV compared to 8.5% for the rural participants (*p* = 0.296). The drugs taken the most were antihypertensives, pain killers such as ibuprofen and paracetamol, antibiotics, and antiretroviral drugs (ARV). High consumption of alcohol (several times a week) was more than double as common among the urban participants than the rural participants (17.6% vs. 7.1%, respectively, *p* = 0.001). Engaging in orogenital sex was uncommon but more common among urban participants than among rural participants (13.3% vs. 4.3%, respectively, *p* = 0.003). The number of orogenital sex partners was also higher in urban participants than in rural participants (*p* = 0.001).

**Table 1:** Distribution of study participants according to habitat

Variables	Urban, *N* (%)	Rural, *N* (%)	Significance
**Gender**			0.026
Man	93 (49.5)	81 (38.4)	
Woman	95 (50.5)	130 (61.6)	
**Age**			0.787
Young adults < 40 years	109 (58.0)	124 (59.0)	
Middle adults 40–59 years	57 (30.3)	58 (27.6)	
Old adults 60 + years	22 (11.7)	28 (13.3)	
**Education**			**< 0.001**
Incomplete primary school or less	53 (28.2)	96 (45.5)	
Completed primary school or more	135 (71.8)	115 (54.5)	
**HIV status**			0.296
Negative	145 (77.1)	158 (74.9)	
Positive	21 (11.2)	18 (8.5)	
Unknown status	22 (11.7)	35 (16.6)	
**Smoking**			0.363
No	115 (61.2)	143 (67.8)	
Ex-smoker	18 (9.6)	15 (7.1)	
Yes	55 (29.3)	53 (25.1)	
**Smokeless tobacco (snuffing)**			0.199
No	173 (92.0)	186 (88.2)	
Yes	15 (8.0)	25 (11.8)	
**Alcohol Use**			**0.001**
Never/seldom	110 (58.5)	154 (73.0)	
Once a month	14 (7.4)	22 (10.4)	
Once a week	31 (16.5)	20 (9.5)	
Several times a week	33 (17.6)	15 (7.1)	
**Ever engaged in sexual intercourse**			0.458
Yes	182 (96.8)	206 (97.6)	
No	6 (3.2)	4 (1.9)	
I do not know	0 (0)	1 (0.5)	
**Number of lifetime sex partners**			0.469
0	6 (3.2)	4 (1.9)	
1–2	100 (53.2)	132 (62.6)	
3–4	37 (19.7)	32 (15.2)	
5–6	17 (9.0)	17 (8.1)	
7–8	8 (4.3)	5 (2.4)	
> 8	20 (10.6)	21 (10.0)	
**Ever engaged in orogenital sex**			**0.003**
Yes	25 (13.3)	9 (4.3)	
No	162 (86.2)	202 (95.7)	
I do not know	1 (0.5)	0 (0)	
**Number of lifetime orogenital sex partners**			**0.001**
0	162 (86.2)	202 (95.7)	
1–2	20 (10.6)	6 (2.8)	
3–4	5 (2.7)	0 (0.0)	
5–6	0 (0.0)	2 (0.9)	
> 8	1 (0.5)	1 (0.5)	

### HPV in the oral cavity

Three out of 399 participants were HPV positive with one single infection. The HPV strains identified were HPV16, HPV35 and HPV45. One study participant was seropositive for HIV. None of the three HPV-positive patients had oral mucosal lesions. One of the three participants was from an urban area and two of them were males. Their ages ranged from 32 to 57 years. The reported number of sex partners were 1–2, 3–4, and > 8, respectively, whereas two out of three reported previous orogenital sex partners.

## Discussion

Only three subjects in this study tested positive for HR-HPV, indicating that oral HPV prevalence in Zambia is low despite the high prevalence of cervical HPV infections and incidence of cervical cancer in the country [12, 13]. The low prevalence of oral HPV could be explained by the small number of participants engaging in orogenital sex. The reported prevalence of orogenital sex practice in the general population varies between studies from sub-Saharan Africa, i.e., ranging from 3.0 to 47.2% [14]. The small percentage of participants engaging in orogenital sex (8.5%) in the present study contrasts sharply with our previous study of individuals living with HIV in Rwanda, where 82% of men and 84% of women reported having engaged in orogenital sex and where the prevalence of oral HPV was 10–12% [10]. The low frequency of participants could also depend on the social stigma of engaging in orogenital sex and that the act is against the law in Zambia. Social stigma related to orogenital sex may affect not only willingness to report sexual practice but also the habit to seek health services, hindering the interventions focused on vulnerable populations. Of note, all three HPV positive cases reported having multiple lifetime sex partners (> 8). Orogenital sex and alcohol consumption were more common in the urban population than the rural population. Studies show that condom use may protect against orogenital HPV transmission [15]. We presently did not examine condom use among the study participants, however, the connection between alcohol consumption and risky sexual behavior including not using condom is well known [16].

We used buccal swabs to assess the presence of HPV from the buccal mucosa with the same technique and methodology as we used in our previous study in Rwanda [10]. HPV can also be tested by oral rinse [17, 18]. Oral rinse has the disadvantage of collecting sample both from the oral cavity and the pharyngeal region and it is not possible to isolate sampling from the oral cavity solely with this technique. In South Africa, oral rinse yielded more viral DNA materials than oral swabs in a study to assess HPV prevalence using both methods [19] and use of cyto-brush revealed retrieval of more positive cases of HPV [20]. Since the prevalence of HPV in the oral cavity was the focus of the study, oral swabs were used in this investigation.

The inclusion of rural and urban clusters in the study sample in the proportion of 56.7% and 43.3%, respectively, is based on the National statistics data of 2019. This balances the social and culture factors in the Zambian population. However, the studied sample size of 399 participants, determined by the population formular that considered the population proportion of 50%, could still be small and probably another reason for the small prevalence of HPV. This reason combined with stigma related to orogenital sex, calls for future longitudinal study regarding oral HPV infection prevalence.

## Conclusions

Despite the presence of many risk factors for contraction of HPV, only three study participants had presence of HR-HPV in the buccal mucosa. Of note, orogenital sex practice was uncommon in the Zambian population where all three participants with present HPV reported a high number of sex partners. Urban participants were more commonly engaged in sexual risk behaviors, including having more orogenital sex partners and consuming more alcohol than rural participants.

## Electronic Supplementary Material

Below is the link to the electronic supplementary material.

Supplementary Material 1

## Data Availability

Additional data can be obtained by writing a request to the Institute of Clinical Sciences Sahlgrenska Academy at the University of Gothenburg, Medicinaregatan 3 A SE-413 90 Göteborg, Sweden. Email: klinvet@gu.se.

## Declarations

### List of unbiased reviewers

1. Professor David Ngassapa, Muhimbili University of Health Sciences and Allied Health, Tanzania, email: dngassapa@yahoo.com. 2. Professor Mohammed Razzaque, Lake Erie College of Osteopathic Medicine, USA, email: mrazzaque@lecom.edu. 3. Professor Joakim Dillner, Center for Cervical Cancer Elimination, Department of CLINTEC, Karolinska Institutet, F46, S-14186, Stockholm, Sweden, email: Joakim.Dillner@ki.se. 4. Professor Gloria Sanchez, Grupo Infección y Cáncer, Facultad de Medicina, Universidad de Antioquia, Medellín, Colombia, email: gloria.sanchez@udea.edu.co. 5. Adriane Kamulegeya, PhD, School of Dentistry, Makerere University College of Health Sciences, Kampala, Uganda, email: adrianek55@gmail.com. 6. Ola Forslund, Associate Professor, Division of Medical Microbiology, Lund University, Lund, Sweden, email: ola.forslund@med.lu.se.
