# Supplementary material for: Oral human papillomavirus infections in Zambian Rural and Urban residents-a community cross-sectional study
Source: BMC Oral Health. 2024 Dec 22;24:1540. doi: 10.1186/s12903-024-05312-4 (PMC11663321; doi:10.1186/s12903-024-05312-4)
Supplement: Supplementary file 1 — Supplementary Material 1 [file 12903_2024_5312_MOESM1_ESM.pdf]

DATA COLLECTION TOOL (QUESTIONNAIRE)

ANNEX A: **Prevalence in East Africa of Human Papillomavirus in the Head and Neck Area and Importance for the Development of Premalignant Lesions and Cancer**

This questionnaire is part of a research project aiming at investigating how common premalignant lesions and cancer in the head and neck area is in Zambia. Moreover, we want to assess the correlation with viral infections and development of cancer. This project is being implemented in the context of the collaboration between Copperbelt University, Ndola (represented by Dr. Chrispinus Mumena; mumenac@gmail.com) and University of Gothenburg in Sweden (represented by Associate Professor Daniel Giglio - daniel.giglio@pharm.gu.se; Principal Investigator). Participants will be given full information on the study (verbally and in writing). The participation in this study is voluntary. Participant identification will be kept anonymous and participants will be given a unique code linking them to their individual questionnaire. Those participants' codes will only be known to the research group and will not be given to anyone outside of the research group.

Questionnaire identification number: | | | | | | | | |

Participant Identification number in the study: | | | | | | | | |

Interviewer Name: ____________________

Interview Date: ____________________

I will ask some questions that will be recorded in this notebook. I must say that everything you answer will be strictly confidential, and the information gathered from you will be used only in scientific reports, without personal identification.

1. BACKGROUND INFORMATION

1A. What is your age? ……………….

1B. Gender

A. Male ☐

B. Female ☐

1C. What is your highest educational level attained?

A. None ☐

B. Incomplete primary school ☐

C. Complete primary school ☐

D. Secondary school incomplete ☐

E. Complete Secondary school ☐

F. Incomplete University ☐

G. Complete University ☐

H. Others (Specify)..

1D. Occupation

A. Farming ☐

B. Civil servant ☐

C. Business ☐

D. Community health worker ☐

E. Unemployed ☐

F. Others (specify) …………………………………………………………………………………

1E. Currently, Marital status?

A. Is married ☐

B. Has a male partner but not married ☐

C. Has a female partner but not married ☐

D. Is separated/divorced ☐

E. Is widow ☐

F. Is single (never been married or lived with a partner) ☐

2. MEDICAL HISTORY

2A. Have you or had any serious diseases in the past?

A. Yes ☐

B. No ☐

If, yes, please specify!

Cardiovascular disease ☐

Cancer ☐

Rheumatic disease ☐

Other serious disease ☐

2B. Do you take any medication regularly?

A. Yes ☐

B. No ☐

Conventional drugs: ………………………………………………………………………

Traditional drugs: ……………………………………………………………….

2C. Do you have any allergy/hypersensitivity?

A. Yes ☐

B. No ☐

If yes, please specify! ……………………………………………………

2D. Type of diet? (Tick more than according to the diet you eat)

A. Vegetarian ☐

B. Non vegetarian ☐

C. Spicy ☐

D. Non spicy ☐

E. Unknown ☐

2E. Do you have bowel disturbances or pain?

A. Yes ☐

B. No ☐

If yes, please specify ……………………………………………………….

2F. Do you have any weight loss?

A. No ☐

B. Yes ☐

C. I don´t know ☐

3. HABITS

3A. Are you a smoker or ever smoked?

A. No, I have never been a smoker ☐

B. Yes, I am currently a smoker ☐

C. I am an ex-smoker ☐

3B. If you are or have been a smoker-What kind of tobacco?

A. No, I have never been a smoker ☐

B. Filter cigarettes ☐

C. Non filter cigarettes ☐

D. Powder tobacco for snuffing ☐

E. Powder tobacco for dipping ☐

F. Dry tobacco leaves for chewing ☐

G. Shisha (water-pipe tobacco) ☐

H. Pipe tobacco without water ☐

I. Reversed smoking ☐

3C. Do you use smokeless tobacco?

A. No ☐

B. Dipping ☐

C. Snuffing ☐

D. Chewing ☐

3D. Have you used smokeless tobacco?

A. No ☐

B. Dipping ☐

C. Snuffing ☐

D. Chewing ☐

3E. How often do you drink alcohol

A. Never/seldom ☐

B. Once a month ☐

C. Once a week ☐

D. Several times a week ☐

3F. How often do you use any of the following (cigarettes, cigars, a pipe, chewing/dipping tobacco, snuffing)?

A. Every day ☐

B. Once a week ☐

C. Several times a week ☐

D. Several times a month ☐

3G. How often do you eat wheat (wheat bread, sandwich, wheat muffin/donut, pizza, pasta)?

A. Every day ☐

B. Every week ☐

C. A few times a month ☐

D. more seldom or never ☐

4. SEXUAL AND REPRODUCTIVE LIVES

We are currently examining virus that can be transmitted through sexual contact. Therefore, we would like to ask you some questions about your sexual and reproductive lives

4A. Have you ever had sexual intercourse?

A. Yes ☐

B. No ☐

4B. At which age in years did you have your first sexual intercourse?

…………………………………………………………………………

4C. How many sexual partners have you had in your life?..

A. 0 partners ☐

B. 1-2 partners ☐

C. 3-4 partners ☐

D. 5-6 partners ☐

E. 7-8 partners ☐

F. >8 partners ☐

4D. Have you ever been engaged in orogenital sex?

A. Yes ☐

B. No ☐

C. I don´t know ☐

4E. With how many partners have you been engaged in orogenital sex?

A. 0 partners ☐

B. 1-2 partners ☐

C. 3-4 partners ☐

D. 5-6 partners ☐

E. 7-8 partners ☐

F. >8 partners ☐

4F. If female: Have you had a Pap smear test?

A. Yes ☐

B. No ☐

C. I don´t know ☐

4G. Do you know your HIV status?

A. Non-infected (negative) ☐

B. Infected (positive) ☐

C. I don´t know ☐

4H. Are you under antiretroviral treatment?

A. Yes ☐

B. No ☐

5. ORAL STATUS

5A. Do you have any known mucosal lesions?

A. Yes ☐

B. No ☐

C. I don´t know ☐

5B. If yes, when was your mucosal lesion first noted?

A. Less than 2 weeks ago ☐

B. 2 weeks to 6 weeks ago ☐

C. More than 6 weeks ago ☐

5C. Which symptoms do you have, related to your mucosal lesion?

A. None ☐

B. Discomfort during eating/speaking ☐

C. Pain ☐

D. Discomfort and pain ☐

E. Others……………………………………………………………………………………

5D. How severe are your symptoms right now (1-10)?

VAS (1=minimum pain-10=maximum):……………………….

5E. How severe is your pain at maximum (1-10)?

VAS (1=minimum pain-10=maximum):…………………………

5F. Have you been given any treatment for your mucosal lesion?

A. Yes ☐

B. No ☐

If yes, please specify **………………………..**

6. HIV

6A. Do you allow us to contact your physician for information on your HIV staging, CD4 counts and viral load?

A. Yes ☐

B. No ☐

Name of your physician and clinic………..………………………

Thank you for your time and effort!

To be completed by a physician from patient file

Patient's HIV Clinical staging………

6B. CD4 + Count (cells/dl)

A. < 200 ☐

B. 2 0 0 -500 ☐

C. > 500 ☐

6C. HIV viral load (copies/ml)

A. < 400 ☐

B. 400-5000 ☐

C. >5000 ☐

Thank you for your time and effort!

7. ORAL STATUS OF PATIENTS

Filled in by researcher at examination ***(Key: use X=missing tooth, Black dot=Filled tooth, v=decayed tooth).***

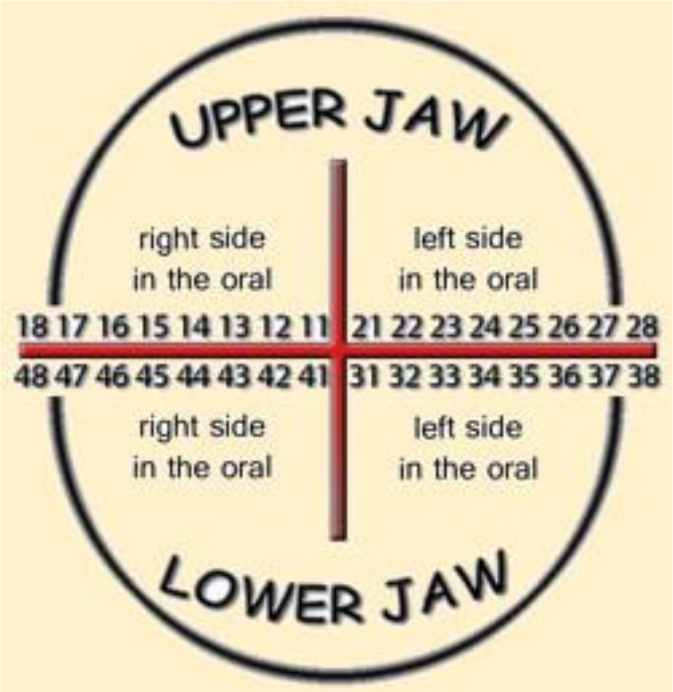

7A. Complete number of teeth

A. **Yes** ☐

B. **No** ☐

If No; number of missing teeth…………………………….

Indicate in the figure! ***Place an X on the missing tooth.***

7B. Teeth with caries at visual inspection*:* ………… ***(Eg. Use black dot for carious tooth on the chat)***

7C. Degree of gingival inflammation ***(use index teeth 16, 26, 36, and 46 to measure pocket depth)***

A. No ☐

B. Mild ☐

C. Moderate ☐

D. Severe ☐

7D. Teeth mobility *(1-3)* …………………………, pocket depth for index teeth …………………

7E. Mucosa status

A. No abnormal growth ☐

B. Non healing Ulceration (Aphthous, herpitic, traumatic, other…………………..) ☐

C. Leukoplakia (white) ☐

D. Erythoplakia (Red) ☐

E. Erythroleukoplakia (red and white) ☐

F. Other, ……………………………………………………………………………………

7F. Mucosal lesion – disease history

………………………………………………………………………………………

7G. Mucosal lesion – status…………………………………………………………………..

7H. Oral mucosal lesion site

A. Mucosa- Labial/buccal/palatal/gingiva/alveolar ☐

B. Tongue-Dorsal/ventral/lateral ☐

C. Floor of mouth ☐

D. Edentulous areas ☐

E. Salivary glands-Parotid/sublingual/submandibular ☐

F. Pharynx ☐

G. Neck-lymph nodes ☐

Diagnosis…………………………………………………………

Provisional clinical diagnosis?…………..

Histopathological

diagnosis?……………………………………………………………………………….

Definitive clinical diagnosis?...............…………………..
